# Development of mass media resources to improve the ability of parents of primary school children in Uganda to assess the trustworthiness of claims about the effects of treatments: a human-centred design approach

**DOI:** 10.1186/s40814-019-0540-4

**Published:** 2019-12-29

**Authors:** Daniel Semakula, Allen Nsangi, Matt Oxman, Sarah Ellen Rosenbaum, Andrew David Oxman, Astrid Austvoll-Dahlgren, Claire Glenton, Simon Lewin, Margaret Kaseje, Angela Morelli, Atle Fretheim, Nelson Kaulukusi Sewankambo

**Affiliations:** 1 College of Health Sciences, Makerere University, Kampala, Uganda; 2 University of Oslo, Oslo, Norway; 3 Centre for Informed Health Choices, Norwegian Institute of Public Health, Postboks 222 Skøyen, 0213 Oslo, Norway; 4 Health Systems Research Unit, South African Medical Research Council, Cape Town, South Africa; 5 Great Lakes University of Kisumu, Kisumu, Kenya; 6 Infodesignlab, Oslo, Norway

**Keywords:** Human-centred design, Intervention-design, User testing, User experience, Mass media, Critical thinking, Critical appraisal, Health education

## Abstract

**Background:**

Claims about what we need to do to improve our health are everywhere. Most interventions simply tell people what to do, and do not empower them to critically assess health information. Our objective was to design mass media resources to enable the public to critically appraise the trustworthiness of claims about the benefits and harms of treatments and make informed health choices.

**Methods:**

Research was conducted between 2013 and 2016 across multiple iterative phases. Participants included researchers, journalists, parents, other members of the public. First, we developed a list of 32 key concepts that people need to understand to be able to assess the trustworthiness of claims about treatment effects. Next, we used a human-centred design approach, to generate ideas for resources for teaching the key concepts, and developed and user-tested prototypes through qualitative interviews. We addressed identified problems and repeated this process until we had a product that was deemed relevant and desirable by our target audience, and feasible to implement.

**Results:**

We generated over 160 ideas, mostly radio-based. After prototyping some of these, we found that a podcast produced collaboratively by health researchers and journalists was the most promising approach. We developed eight episodes of the Informed Health Choices podcast, a song on critical thinking about treatments and a reminder checklist. Early versions of the podcast were reportedly too long, boring and confusing. We shortened the episodes, included one key concept per episode, and changed to story-telling with skits. The final version of the podcast was found to be useful, understandable, credible and desirable.

**Conclusion:**

We found many problems with various prototypes of mass media resources. Using a human-centred design approach, we overcame those problems. We have developed a guide to help others prepare similar podcasts.

## Background

We encounter claims about the effects of treatments (any action intended to improve health) all the time. This includes claims about the effects of drugs, surgery and other types of “modern medicine”; claims about lifestyle changes, such as changes to what you eat or how you exercise; claims about herbal remedies and other types of “traditional” or “alternative medicine”; claims about public health and environmental interventions; and claims about changes in how healthcare is delivered, financed and governed. New treatment claims are made every day in the mass media.

While some claims are trustworthy, many are not, and the trustworthiness of claims found in the mass media frequently is not adequately assessed [1–11]. This can affect health behaviours and healthcare use [12–14]. To make informed choices, people need to be able to assess the trustworthiness of treatment claims. Untrustworthy treatment claims and misinformed decisions about treatments result in wasted resources and unnecessary suffering [15–19]. This is a universal problem, but the consequences are likely to be greater in settings where resources are scarce [20–24].

The Informed Health Choices (IHC) project was established with the aim of developing learning-resources to improve people’s ability to assess the trustworthiness of claims about treatment effects and enable them to make informed decisions about treatments [25]. Our initial focus was on low-income countries where disparities in access to information, education and care are likely to be larger and the consequences of making poorly informed health choices are likely to be greater [26–29]. In the first phase of this work, we developed a list of 32 key concepts that people need to understand in order to be able to assess treatment claims and make informed decisions [30]. The key concepts can help people to recognise treatment claims that have an unreliable basis, understand whether comparisons of treatments are fair and reliable and make informed decisions about treatments. Journalists in Uganda judged the concepts to be relevant to journalists and their audiences and possible for them to learn [31].

The IHC key concepts served as a framework for developing two sets of learning-resources: one for primary schools and one for the mass media in Uganda [32]. The development of the primary school resources is described elsewhere [33], and other potential applications of the key concepts are described in another report [32]. This article describes the development of mass media resources designed to enable people to understand and apply the IHC key concepts to assess the trustworthiness of claims about treatment effects and make informed health choices.

## Methods

**Fig. 1:**
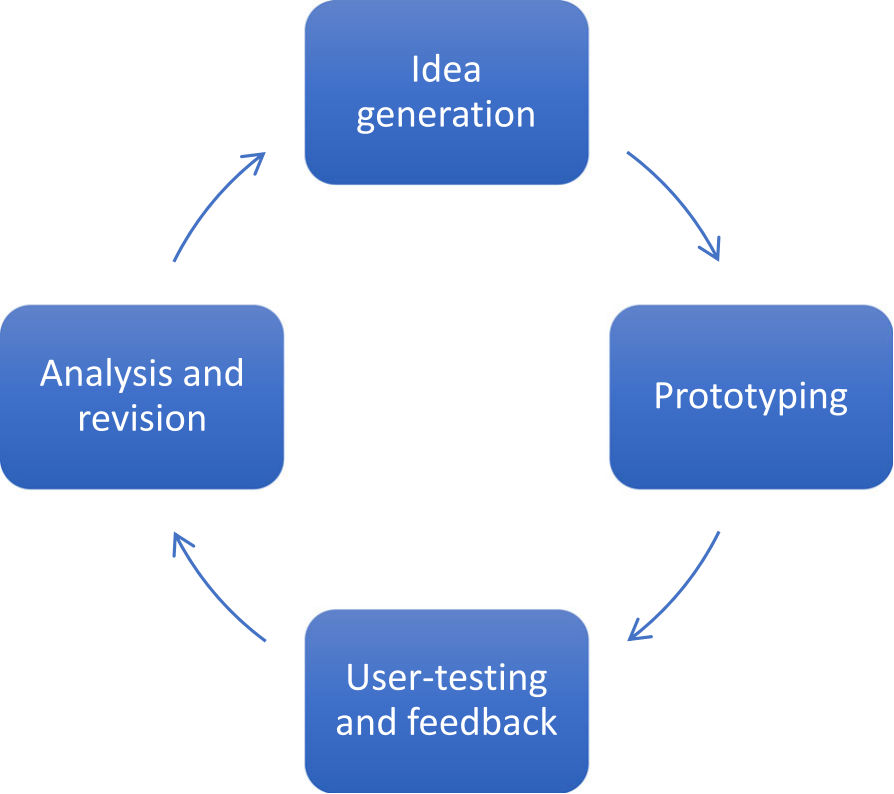
One cycle of a human-centred design process

We used design thinking methods. Design thinking espouses five major steps: (1) empathising to define the problem, (2) defining the problem, (3) ideation, (4) prototyping (experimenting on potential solutions) and (5) testing [34]. We overlaid design thinking with a human-centred design approach [35–38]. This approach is characterised by multiple iterative cycles of development. For simplicity, we have summarised that process into four steps: (1) idea generation, (2) prototyping, (3) user testing and (4) analysis and incorporation of findings (Fig. 1).

### Setting

This project was implemented in Central Uganda. The majority (over 70%) of people live in rural areas only slightly more than 30% have attained at least secondary school education [39].

### Participants

Different participants were involved at different stages as described in Table 1, which presents a chronological descriptive summary of each phase and who participated. Throughout the project, we established and maintained contact with a national advisory panel consisting of officials from three government ministries (health, education and one concerned with children, labour, gender and social development). We also established and maintained contact with a network of teachers, and journalists, editors of Ugandan media enterprises and communication specialists, whose role was to advise on strategies for successful implementation of the project.

**Table 1:** Overview of the methods, key activities and participants in each phase of the development process

Method type/date	Participants	Description of key activities
Idea generation and exploratory prototypes		
Review of existing resources February 2013 to September 2014	The research team (AA, AM, AN, AO, CG, DS, SL, SR)	We searched for and reviewed existing mass media resources that teach the key concepts.
Idea generation workshop (participatory collaboration) February 2013	Researchers, teachers and journalists from Indonesia, Nepal, Norway, Uganda and the UK	At the 3-day kick-off meeting for the project, the research team together with invited teachers and journalists (18 people) discussed which concepts to focus on and brainstormed about potential resources.
Prioritisation of key concepts (participatory collaboration) August 2013	The journalists’ network in Uganda (25 journalists) [17]	At a 3-day workshop, the journalists assessed the relevance of a list of 32 key concepts to journalists and their audiences.
Prototyping workshop (facilitation and non-participatory observation) September 2013	The journalists’ network in Uganda, (25 journalists)	This was a full-day workshop at which journalists brainstormed and created prototypes.
Idea generation meetings and prototyping (participatory collaboration) October 2013 to October 2014	The research team (AA, AM, AN, AO, CG, DS, LN, MK, MO, NS, SL, SR)	We had a series of meetings during which we brainstormed. One idea was a service that would provide structured press releases, including application of the key concepts to a treatment claim. We prototyped one press release. Another was a wire service that would produce short stories that would explain key concepts to readers and listeners in the context of news about a specific treatment claim. We prototyped two examples of stories produced by such a news service; one as a print story and one as a radio programme.
Focus group feedback (focus group discussion and semi-structured interviews) October 2014	Four media editors, a journalist and a health communication specialist. Four random members of the public	Structured press releases: The participants read the press release, and then provided feedback.
Focus group feedback (focus group discussion and semi-structured interviews) October 2014	Four media editors, a journalist and a communication specialist. Four random members of the nonacademic public.	News service: The participants read or listened to each of the stories and then provided feedback. Following this, we interviewed three of the participants of the focus group discussion and each of the four members of the public
Semi-structured interviews October 2014	Four members of the general public	The participants listened to and read the prototypes of the messages and provided feedback about the news service. Any problems identified were noted and followed up.
Analysis of findings and idea generation October 2014	The research team (AA, AM, AN, AO, CG, DS, LN, MK, MO, NS, SL, SR)	We reviewed the feedback on the news service prototype and generated ideas to address the problems that we identified.
Version 1. The Health Choices (radio) programme (v1)		
V1 Development of a prototype October 2014 to April 2015	The research team (AA, AM, AN, AO, CG, DS, MO, SL, SR)	We outlined plans for a series of what we initially thought of as a radio programme and prepared prototypes of two versions of the first episode; one using an interview format and one using a story format.
V1 User testing in Uganda, (semi-structured interviews) April 2015	Two health journalists and four other members of the nonacademic public	Two versions of prototype 1 were tested in sequence. First, the participants listened to the first version of the prototype (1a) and provided early feedback. We then user-tested an alternative prototype (1b) of the same contents as the first with a story-based theme. Prototype 1b was partly based on early feedback from the testing of prototype 1a.
V1 Analysis and idea generation for V2 May 2015	The research team (AA, AM, AO, CG, DS, MK, MO, NS, SL, SR)	We analysed the feedback and discussed findings from the user testing and feedback on the first version of the IHC podcast and generated ideas to address the problems that were identified.
Version 2. The IHC podcast (v2)		
Interviews with parents to identify relevant claims (semi-structured interviews) March–April 2015	30 parents	We interviewed parents to identify health conditions and treatments that were relevant to them.
V2 Development of the second complete prototype June to August 2015	The research team (AA, AM, AN, AO, CG, DS, MK, MO, NS, SL, SR) Radio producer, actors, journalists and parents of primary school children	We prepared a series of nine episodes targeted at the parents of primary school children in Uganda. MO prepared a script for each episode, which was edited by DS and AO, and other team members provided feedback. A professional radio producer and actors produced the episodes.
V2 User testing and piloting in Uganda (semi-structured interviews) September to December 2015	28 parents and 7 research assistants	28 parents listened to the podcast. We interviewed them after they listened to each episode. With the help of the parents and research assistants, we also piloted a method for delivering the podcast to the parents in areas where they live and work, collecting feedback on the method and technologies used.
V2 Analysis and idea generation for V3 December 2015 to January 2016	The research team (AA, AM, AN, AO, CG, DS, LN, MK, MM, MO, NS, SL, SR) Radio producer, journalists and parents.	We entered the findings into a Google spreadsheet. For each finding, AN, AO, DS, MM, MO and SR coded its importance (very important, important or less important); whether it was a problem, an idea or positive feedback; and whether it applied to the entire podcast, a specific episode or was a repeat of a previous finding. The findings were summarised for the research team and the major findings and plans for the third version, and the community trial were discussed and agreed.
Version 3. The final IHC podcast (v3)		
V3 Development of the final podcast January to March 2016	The research team (AA, AM, AN, AO, CG, DS, LN, MK, MM, MO, NS, SL, SR) Radio producer, actors, musicians, parents of primary school children, other members of the general public	MO prepared new scripts, which were edited by DS and AO. Other team members provided feedback. DS translated scripts to Luganda. DS, AN, AO prepared the lyrics to the theme song. Other members provided feedback. A professional musician was commissioned to edit the lyrics and produce the song. A professional radio producer and actors produced the episodes. DS, AN, AO and MO reviewed the produced episodes and suggested edits to the production.
Review of existing resources February 2013 to September 2014	The research team (AA, AM, AN, AO, CG, DS, SL, SR)	We searched for and reviewed existing mass media resources that teach the key concepts.
Idea generation workshop (participatory collaboration) February 2013	Researchers, teachers and journalists from Indonesia, Nepal, Norway, Uganda and the UK	At the 3-day kick-off meeting for the project, the research team together with invited teachers and journalists (18 people) discussed which concepts to focus on and brainstormed about potential resources.
Prioritisation of key concepts (participatory collaboration) August 2013	The journalists’ network in Uganda (25 journalists) [17]	At a 3-day workshop, the journalists assessed the relevance of a list of 32 key concepts to journalists and their audiences.
Prototyping workshop (facilitation and non-participatory observation) September 2013	The journalists’ network in Uganda, (25 journalists)	This was a full-day workshop at which journalists brainstormed and created prototypes.
Idea generation meetings and prototyping (participatory collaboration) October 2013 to October 2014	The research team (AA, AM, AN, AO, CG, DS, LN, MK, MO, NS, SL, SR)	We had a series of meetings during which we brainstormed. One idea was a service that would provide structured press releases, including application of the key concepts to a treatment claim. We prototyped one press release. Another was a wire service that would produce short stories that would explain key concepts to readers and listeners in the context of news about a specific treatment claim. We prototyped two examples of stories produced by such a news service; one as a print story and one as a radio programme.
Focus group feedback (focus group discussion and semi-structured interviews) October 2014	Four media editors, a journalist and a health communication specialist. Four random members of the public	Structured press releases: The participants read the press release, and then provided feedback.
Focus group feedback (focus group discussion and semi-structured interviews) October 2014	Four media editors, a journalist and a communication specialist. Four random members of the nonacademic public.	News service: The participants read or listened to each of the stories and then provided feedback. Following this, we interviewed three of the participants of the focus group discussion and each of the four members of the public
Semi-structured interviews October 2014	Four members of the general public	The participants listened to and read the prototypes of the messages and provided feedback about the news service. Any problems identified were noted and followed up.
Analysis of findings and idea generation October 2014	The research team (AA, AM, AN, AO, CG, DS, LN, MK, MO, NS, SL, SR)	We reviewed the feedback on the news service prototype and generated ideas to address the problems that we identified.
Version 1. The Health Choices (radio) programme (v1)		
V1 Development of a prototype October 2014 to April 2015	The research team (AA, AM, AN, AO, CG, DS, MO, SL, SR)	We outlined plans for a series of what we initially thought of as a radio programme and prepared prototypes of two versions of the first episode; one using an interview format and one using a story format.
V1 User testing in Uganda (semi-structured interviews) April 2015	Two health journalists and four other members of the nonacademic public	Two versions of prototype 1 were tested in sequence. First, the participants listened to the first version of the prototype (1a) and provided early feedback. We then user-tested an alternative prototype (1b) of the same contents as the first with a story-based theme. Prototype 1b was partly based on early feedback from the testing of prototype 1a.
V1 Analysis and idea generation for V2 May 2015	The research team (AA, AM, AO, CG, DS, MK, MO, NS, SL, SR)	We analysed the feedback and discussed findings from the user testing and feedback on the first version of the IHC podcast and generated ideas to address the problems that were identified.
Version 2. The IHC podcast (v2)		
Interviews with parents to identify relevant claims (semi-structured interviews) March–April 2015	30 parents	We interviewed parents to identify health conditions and treatments that were relevant to them.
V2 Development of the second complete prototype June to August 2015	The research team (AA, AM, AN, AO, CG, DS, MK, MO, NS, SL, SR) Radio producer, actors, journalists and parents of primary school children	We prepared a series of nine episodes targeted at the parents of primary school children in Uganda. MO prepared a script for each episode, which was edited by DS and AO and other team members provided feedback. A professional radio producer and actors produced the episodes.
V2 User testing and piloting in Uganda (semi-structured interviews) September to December 2015	28 parents and 7 research assistants	28 parents listened to the podcast. We interviewed them after they listened to each episode. With the help of the parents and research assistants, we also piloted a method for delivering the podcast to the parents in areas where they live and work, collecting feedback on the method and technologies used.
V2 Analysis and idea generation for V3 December 2015 to January 2016	The research team (AA, AM, AN, AO, CG, DS, LN, MK, MM, MO, NS, SL, SR) Radio producer, journalists and parents.	We entered the findings into a Google spreadsheet. For each finding, DS, AN, AO, MM, MO and SR coded its importance (very important, important or less important); whether it was a problem, an idea or positive feedback; and whether it applied to the entire podcast, a specific episode or was a repeat of a previous finding. The findings were summarised for the research team and the major findings and plans for the third version, and the community trial were discussed and agreed.
Version 3. The final IHC podcast (v3)		
V3 Development of the final podcast January to March 2016	The research team (AA, AM, AN, AO, CG, DS, LN, MK, MM, MO, NS, SL, SR) Radio producer, actors, musicians, parents of primary school children, other members of the general public	MO prepared new scripts, which were edited by DS and AO. Other team members provided feedback. DS translated scripts to Luganda. DS, AN, AO prepared the lyrics to the theme song. Other members provided feedback. A professional musician was commissioned to edit the lyrics and produce the song. A professional radio producer and actors produced the episodes. DS, AN, AO and MO reviewed the produced episodes and suggested edits to the production.

In an early phase of the project, our principal target audiences were “mass media intermediaries”—journalists and editorial news teams. Aiming to reach a broader public through intermediaries, we explored ideas about resources we might develop to support their work, so they could report health stories about treatment claims more critically and informatively. We established a network of 25 Uganda journalists with interest and experience in health reporting, to generate resource ideas and provide feedback on prototypes. These were conveniently selected based on their availability and interest in health reporting and in the project. We recruited them by contacting leaders of three major health journalists’ professional organisations in Uganda (Uganda Science Journalists’ Association, Health Journalists Network of Uganda and Uganda Health Communication Alliance) and the national professional organisation for journalists (Uganda Journalists’ Association). We asked them to avail us with names of journalists and media practitioners involved in health-related reporting. We describe this process in greater detail in another report [31]. We also recruited a group of editors from Uganda media houses, based on recommendations from the journalists in our network and others working in mass media organisations.

In subsequent phases, our focus shifted to creating resources that would target mass media audiences directly instead of through intermediaries. In order to narrow our focus further and to complement the set of learning-resources we were developing for children in primary schools, we defined our target audience as the parents of primary school children in Uganda We recruited parents with children in year five of primary school who, like the journalist network, participated by generating ideas and providing feedback on prototypes and the subsequent versions of the resources.

The research team participated in idea generation, data analysis and prototype refinement. It included researchers with backgrounds in health systems research, journalism, public health, medicine, social sciences and information design. We engaged professional radio presenters, actors, musicians and music producers to help develop the final versions of the mass media resources.

### Procedures

**Fig. 2:**
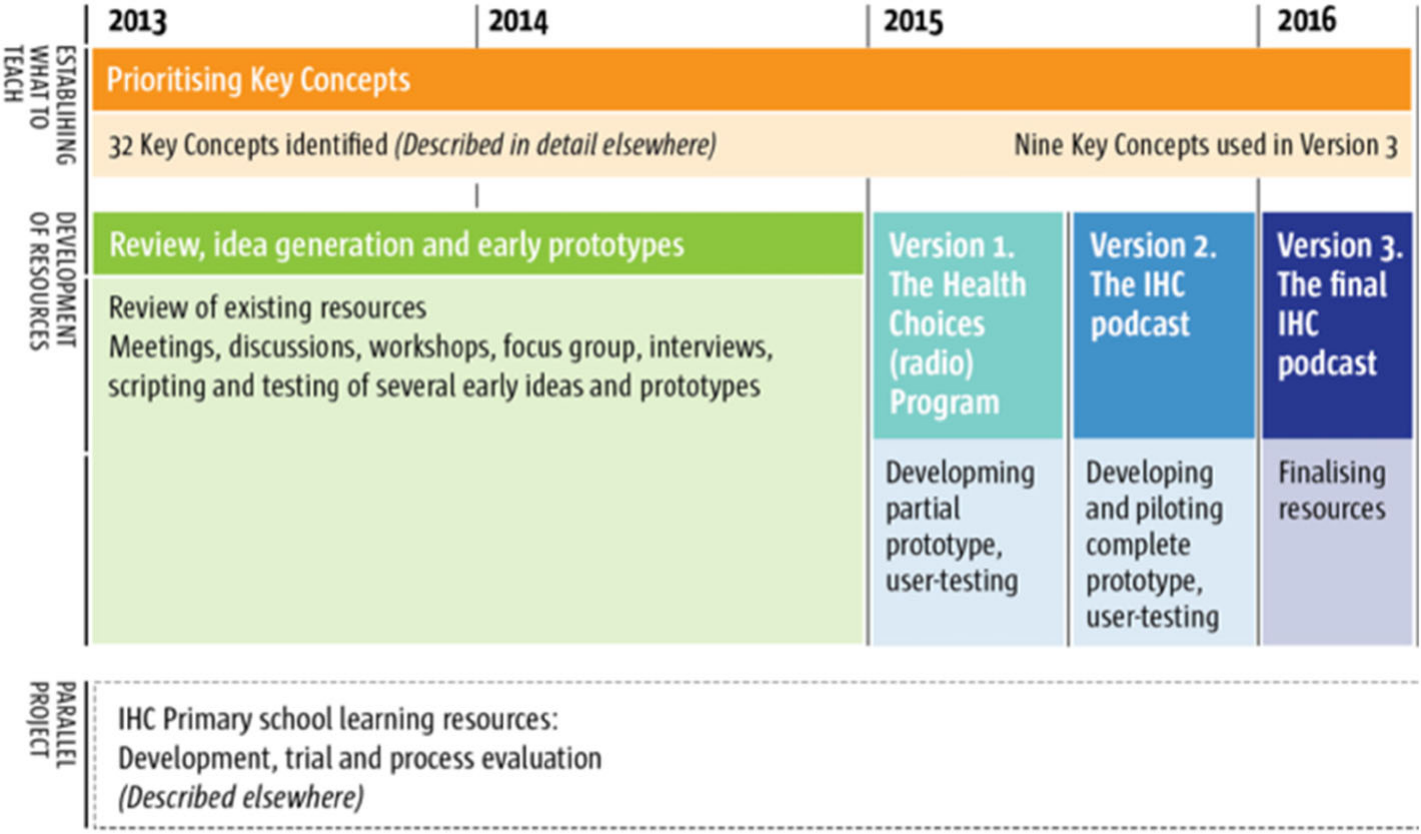
Development flow chart for the IHC mass media resource. This is a summary of the development process as it unfolded across the timescale of the project

The development work entailed five phases: (1) review of existing resources and prioritisation of key concepts, (2) idea generation and exploratory prototypes, (3) version 1 of what became the IHC podcast, (4) version 2 of the IHC podcast and (5) version 3 of the IHC podcast (Fig. 2).

### Review of existing resources and prioritisation of key concepts

We searched for and reviewed existing mass media and other resources designed to improve public understanding of health information and their ability to assess the trustworthiness of information about claims of the effects of treatments in the mass media. We searched relevant databases for published literature and contacted researchers in relevant fields [40, 41]. We held workshops with key stakeholders such as journalists to set priorities for key concepts for which we should develop resources [31].

### Idea generation

To generate new ideas for resources to be developed, we conducted brainstorming sessions both within our research team and with the journalists network [42]. In addition, we conducted a 1-day workshop with journalists in which we explained to them the idea and asked for their contributions (Additional file 1).

### Prototyping

We collected all of the ideas that we generated in a spreadsheet from which we selected ideas for prototyping (Additional file 1). The criteria used to decide which ideas to develop resources for is summarised in Table 2 below.

**Table 2:** Criteria for deciding on prototypes to develop

Criterion	Description
Contextual appropriateness	The idea should align and be seen to align with the cultural, political norms and expectations in the context where the intervention is to be developed
Feasibility	The ideas can potentially be developed easily and practically with resources that are readily available in the context in which it is to be developed.
Cost	Developing the idea should have a reasonable cost, given the available budget and the context in which the resources would be developed.
Flexibility	Resources developed using the idea could be used or changed in different ways, e.g. by inserting or removing parts without causing a lot of problems.
Replicability	It should be possible to replicate the development of the resources without losing important attributes and information
Transferability	To the extent possible, the resources or parts of the resources developed from the idea should be able to be used in other contexts without much difficulty.
Self-reliant	Using the resources developed from the idea should depend on having other resources, e.g. regular support from health workers or teachers.
Scalability	It should be possible to use the resources on a wider scale

Working with information designers and journalists, we converted written descriptions of ideas into prototypes. For example, for ideas about print-based news stories, we wrote actual news stories based on claims about treatment effects in the media, while for ideas of radio programmes we produced and recorded a version of a radio programme. These prototypes were then shared with potential users, journalists and members of our research team for user testing and feedback.

### User testing and feedback

We varyingly used a combination of concurrent and retrospective think aloud and retrospective probing methods of formative usability testing to monitor people’s understanding of the content of our products along the development pathway. In the concurrent think aloud method, testers are encouraged to verbalise their thoughts as they test a service while in retrospective think aloud method, they recall their thoughts and can relay them in post-use interviews and discussions [43]. For instance, to collect feedback about a news service idea, we presented an example of a news story that might come from such a service to a group including editors, a journalist and a communication specialist. As they read it, they made notes on the articles, marking areas where they had comments and sometimes voicing those out in real time. In addition, we used prompts to elicit discussion and feedback about the specific example as well as the news service concept itself and took notes. In addition to the focus group discussion, we interviewed individual participants using a semi-structured interview form, recorded the interviews and took notes.

In the later phases when we had created prototypes of the podcast, we carried out user testing with people in our target audience. User testing is a process of formative evaluation of a product or service that involves observing a person using a product or service and obtaining feedback during or shortly after they interact with it [35, 36].

To assess changes in user experiences along the development pathway, we interviewed user-test participants serially at different stages of the design process. This allowed us to monitor trends in perceptions about the resources in development. We monitored for changes in perceptions regarding the relevance, value, usefulness and other facets of the user-experiences honeycomb framework. We were able to tell when and how perspectives changed with every new prototype and version of the final resources.

We entered the feedback both from user-test participants and from members of the research team into Google spreadsheets. At least two researchers from the IHC team working independently coded each observation for each version of the IHC podcast based on the importance of the finding and its implications for changes to the podcast. The coding was combined in a single spreadsheet, discussed by the coders and consensus was reached (Table 3). Based on these findings, we generated a list of problems and suggestions for changes. We discussed major problems and brainstormed solutions to those problems with the rest of the IHC team. After agreeing on the changes that we would make, we created new prototypes to be user-tested and the design cycle continued until we came up with a final product. We used three main criteria to guide when to end the development iterations:

1. Most of the problems of importance identified in previous prototypes have been addressed as evidenced by their absence in subsequent prototypes and more positive feedback on areas where those problems were.
2. Convergence of responses pertaining to understanding of the content
3. No new major problems have been identified in new prototypes as a result of changes made to previous ones.

**Table 3:** Coding of the importance of feedback for the users’ experience

Category	Description
Highly important problem	A problem with the resources that must probably be addressed for the resources to be effective
Important problem	A problem with the resources that should probably be addressed for part of the resources to be effective
Problem	A superficial problem with the resources
Highly important positive feedback	Positive response that probably should inspire in changes to the resources
Important positive feedback	Praise that maybe should inspire changes to the resources
Positive feedback	Praise that validates the resources as they are
Highly important idea	An idea that probably should inspire changes to the resources
Important idea	An idea that maybe should inspire changes to the resources
Idea	An idea that probably should not inspire changes to the resources

During the development of the second version of the IHC podcast, we discussed methods for delivering the podcast to our target audience. The feasibility of these methods was assessed in a pilot exercise using the completed episodes of version 2. The experiences of research assistants and parents were captured using a semi-structured interview form. Findings from this process generated information about the practical requirements for conducting a community-based randomised trial evaluating the effectiveness of the podcast [44] and informed our next steps in the development and delivery of version 3 of the IHC podcast.

## Results

### Participants’ characteristics

Members of the journalists’ network included 14 females and 11 males with a median age of 32 years and median years of experience of 5 years. The majority (16 out of 25) worked with private media enterprises while the rest worked with government (8 out of 25) or government-private media partnerships (1 out of 25). Most (18 out of 25) worked in urban areas and had at least tertiary education (23 out of 25).

Parents who participated in the user testing were mainly female (20 out of 28), had a median age of 28 years, had an education level of primary or less (19 out of 28) and were employed in the informal sector running small home-based businesses (25 out of 28).

### Review of existing resources and prioritisation of key concepts

We reviewed a total of 415 eligible studies conducted over the last seven decades. We found that only a handful of the interventions and evaluation tools described included the key concepts that we deem important for people to understand in order to be able to critically appraise the trustworthiness of treatment claims. The key concepts that appeared more frequently in interventions were “Treatments may have beneficial and harmful effects”, “Comparisons of treatments should be fair”, “Like should be compared with like” and “Single studies can be misleading”. We identified a number of different evaluation tools, but only four of these included 10 or more key concepts. None covered all the key concepts. These results are reported in detail elsewhere [40]. A priority-setting exercise we conducted early in the project participants found all six groups of the key concepts to be important, applicable and understandable. The full results from this phase are reported elsewhere [31].

### Idea generation and exploratory prototypes

We initially intended to develop tools for journalists to help them write articles and produce media programmes that would enable the public to think more critically and acquire skills to assess the trustworthiness of claims about the effects of treatments. Through brainstorming sessions with the research team and with journalists, we generated many ideas for doing this. These included the following:

- Practical resources, such as structures for reporting claims about treatment effects, visual aids that could be used to present and explain research evidence, glossaries of health research terms and plain language tools
- Training modules for journalists and journalism students on reporting health research and critically assessing and reporting claims about the effects of treatments
- A journalist network and a researcher network to support journalists in reporting claims about treatment effects

Tools such as these might help to address some barriers to improving reports of treatment claims [45], such as difficulties making health research jargon understandable and access to reliable sources of evidence. However, we decided against these ideas because they would not address important underlying barriers that make it difficult for journalists to report more informatively such as commercialism in the media (the need for journalists to sell stories, which can conflict with providing balanced information) and organisational constraints (such as editors that can be an obstacle to preparing more informative reporting of treatment claims). In addition, we thought that, to the extent that we could develop effective tools for journalists, it was unlikely that they would be widely used outside of a small subset of health journalists, further limiting their impact. Input from the journalist network supported the conclusion that developing tools for journalists would have little if any impact in Uganda.

In the prototyping workshop, the journalists agreed that radio was the best way to reach the broadest audience in Uganda. Most of the prototypes they developed were live talk shows, a format that is popular in Uganda involving health experts as panellists and journalists as moderators. The pros and cons for using radio are summarised in Table 4 below.

**Table 4:** Journalists’ reasons for and against using radio

In favour of using radio	Against using radio
1. Easily accessible to a large section of the public 2. Free of charge to access 3. Can be entertaining 4. Allows flexible use of local languages	1. Audience’s perceived difficulty to tune in to a show consistently at the time a programme is aired 2. Lack of options to pause or replay the radio programmes at will. 3. Unstable access to electricity in some areas 4. The large volume of competing information on radio. 5. The need to use multiple languages. 6. It would be very challenging to get the right people to answer questions as experts on live radio talk shows as health professionals are usually very busy. 7. It is difficult to achieve consistent messaging when running live talk shows

#### Shifting from resources targeting mass media intermediaries to those for the general public

The findings above relating to difficulties associated with developing resources for journalists led us to shift focus from developing resources for supporting media intermediaries to collaborating with them to jointly produce and publish content. We proceeded to develop two prototypes: first, a rapid response service to meet the public’s needs for information about assessing the trustworthiness of claims about the effects of treatments, and then a news wire service to produce short stories for publication in the media (Additional file 2). Co-producing and publishing content with journalists was also seen by journalists and media intermediaries as a nonviable option. Key findings from the feedback we received on these prototypes are summarised in Table 5 below:

**Table 5:** Feedback on early prototypes (rapid response service and news wire service)

Main theme	Specific feedback
Focus on audio messaging	• Focus on audio messages through radio, as this is the most accessible means of mass communication. • Make stories available for listening and download online (e.g. via Facebook, YouTube, Sound Cloud, a project website and iTunes. • Consider a series of features prepared for specific media (e.g. regional radio) rather than a news service.
Narrow down the target audience	• Segment the resources for specific target audiences as it is difficult to develop a single product that appeals to all.
Make the aim and content clearer	• Make it clearer to the audience that we are empowering them to assess claims about the effects of treatments, not assessing the claims for them. • Provide a clear message regarding the trustworthiness of each claim. • Consider using more than one example in the explanations and use claims that are of interest to the target audience. • Repeat important information in each story. • Consider a checklist or a list of reminders for our audience as a quick reference tool. • Use more than one language.
Ensure credibility of the project, content and sender	• Ensure the audience knows that there is a credible organisation behind the project. • Provide more information about the claims and their origins to avoid the audience thinking that we are making the claims. • Ensure that the editors, producers and other “gatekeepers” understand what the project is about.
Additional considerations:	• Train journalists and editors. • Include fact-checking packages with stories. • Promote the project and stories ahead of time in various media.

Based on these findings, we began to explore creating a series of pre-recorded audio messages about assessing the trsutworthiness of claims about the effects of treatments. We decided to develop a programme that closely resembled a live interview talk show. We worked with a multidisciplinary team including journalists, professional actors, editors, health professionals, health researchers and members of our target audience to develop ideas, write scripts and produce a series of pre-recorded audio episodes. A pre-recorded radio show was chosen over a live radio show because of the logistical and technical challenges related to producing live radio shows such as consistency in messaging by presenters and the reliance on media-savvy researchers, skilled moderators, among others. The scripts would be about assessing claims about treatment effects, and recordings could be hosted across multiple electronic media platforms. We initially planned on producing this as a radio programme. Some of the contents of the episodes are outlined in Table 6.

**Table 6:** Claims used in main episodes of IHC Podcast versions 2 and 3

Episode and main lesson/key concept^§^	Claim used in the episode and issues of concern or subject for discussion	The issues or subject for discussion about the claim and reason for inclusion
Episode 1 Most treatments have both good and bad effects (benefits and harms)	“There are herbal medicines that cure malaria and do not have any bad effects.”	The claim that herbal treatments do not have any bad effects is untrustworthy since most treatments can have both good and bad effects. How sure can one be that herbal treatments are indeed without any bad effects?
Episode 2 Knowledge about the effects of treatments requires comparisons	“Zmapp, a new investigational drug in evaluation can cure Ebola Virus Disease”	Zmapp was an investigational drug at the time. Evaluation of Zmapp was not yet complete at the time of production but it was given to some health workers who subsequently improved. Given the information available at the time, how sure could we be that Zmapp cures Ebola Virus Disease?
	“Eating quail eggs can make one very strong.”*	There was no known evaluation at the time comparing taking quail eggs to taking nothing or to anything else, to establish if eating the quail eggs makes one stronger. How sure can one be that eating quail eggs will make one stronger in the absence of any fair evaluation of their effects?
Episode 3 Personal experiences are not a reliable basis for claims about treatment effects	“Putting cooking oil on a burn will heal it since it has worked for someone else before”	The claim was based on someone’s personal experience using cooking oil on burns wounds. How reliable are personal stories (anecdotes) at predicting how treatments will work?
Episode 4 An effect on an outcome may be associated with a treatment, but it may not be the treatment causing the effect to happen	“A lot of women gain weight when they take contraceptive pills.”	This claim was based on the association between women using contraceptives and adding weight. Is it possible that an effect on an outcome could be associated with a treatment when it is not the treatment causing the effect?
Episode 5 How long a treatment has been used or how many people have used it is not a reliable basis for judging the effects of treatments.	“An herbal treatment called ‘kyogero’ stops babies from getting infections because many people have used it for a long time.”	This claim is based on the finding that many people have used the herbal treatment for a long time. Does the finding that many people have used a treatment for a long time mean that the treatment is effective and/or safe?
Episode 6 Opinions of experts can be misleading if they are not based on reliable evidence	According to one expert, “taking some hot pepper will heal stomach ulcers”.	The claim was based simply on what an expert said- an expert opinion. Is it possible that experts can be wrong in their opinions, for example, if they are not based on the best evidence?
Episode 7 Comparisons of treatments should be fair	“Medical male circumcision reduces the chances of acquiring HIV.”	This claim was based on a fair comparison of medical male circumcision to prevent HIV and no circumcision. What are fair comparisons? Do fair comparisons of treatments offer a reliable basis for determining if treatments are effective and/or safe?
	*“Group support treatment is helpful for someone who has depression and HIV because the treatment has been compared with other alternatives and found to be effective.”	This claim was based on a fair comparison of using group support treatment and not using it for people with depression. What are fair comparisons? Do fair comparisons of treatments offer a reliable basis for determining if treatments are effective and/or safe?
Episode 8 Single comparisons of treatments or comparisons with very few people can be misleading	According to findings from a small study: “washing hands with soap does not stop children from getting diarrhoea”.	The claim was based on a single study with very few participants. To what extent can we rely on single studies with very few participants?

*Claims used in version 3 of the IHC podcast in place of the one used in version 2

^**§**^A complete description of the IHC Key concept and their implications can be found in Austvoll-Dahlgren et al. [30]

#### Version 1: the Health Choices radio programme

Because of the problems earlier identified with live radio, we chose to produce a pre-recorded radio programme (Table 7).

**Table 7:** The Health Choices radio programme

The Health Choices radio programme featured a radio show host who interviewed a health researcher and a professor about two treatment claims. For each claim, people from the target audience gave their opinions before and after the trustworthiness of the claim was discussed by the three show participants. To explain the trustworthiness of each claim, the guests (health researcher and professor) applied an IHC key concept to assess the claim and used an analogy to help explain that concept. Then, the best available evidence from a systematic review was presented and used to assess the trustworthiness of the claim. More information was provided about where a listener could access research evidence pertaining to similar claims. Key take-home messages were about how to assess the trustworthiness of treatment claims. We produced two prototypes, both of which can be found here (https://www.youtube.com/playlist?list=PLeMvL6ApG1N2G_aT-nfOI1NAOyF9FzKjb). Each episode had the following: 1. Welcome remarks for the programme and the episode 2. A recap of the previous episode 3. An overview of the episode 4. A skit introducing the first claim 5. Opinions from three people from the target audience about the first claim before listening to an explanation 6. Explanation of the reliability of the first claim applying an IHC key concept 7. A presentation of the findings of a systematic review 8. Opinions from the same three people about the claim after listening to the explanation and evidence 9. Introduction of the second claim and a repetition of steps 5 to 8 10. Conclusion of the episode	

User testing of the first prototype indicated that the detailed explanations were valued, the opinions of members of the public provided authenticity to the programme and the health researcher and professor provided credibility to the programme. However, we identified several problems as outlined below:

### Problems associated with the first prototype: the health choices radio programme

- The first episode was too long (15 min) and the explanations were confusing. There was too much information packed into a single episode, which made it difficult to follow.
- Presenting two claims in one episode created confusion about the take-home messages.
- The programme was not interactive; it had long explanations which made it boring.
- The three members of the audience (in the programme) had very strong opinions about the claims, which were wrong and were sustained after listening to the explanations and the evidence. In addition, some of them introduced new claims about treatment effects when giving their opinions about the claims that we were discussing in the episode.
- The pre-recorded interview sounded unnatural when scripted.

### Incorporating feedback into the redesign of the programme

Based on the above findings, we decided to modify the format of the programme, to shorten each episode, to only include one claim in each episode and not to include the opinions of people from the target audience but to use a skit to introduce the claim to be discussed. The skit was in the form of a story. We produced a new prototype that was 8 min, removed the long introduction, shortened the explanations and the conclusion and only used one claim. User testing with members of our target audience (adult members of the public) indicated that the shorter version was better than the previous version and that the explanation using an analogy and examples was good.

However, listeners still confused the claim and the main message about applying an IHC key concept to assess the trustworthiness of the claim. They also still found the programme boring, and the introduction too long. We therefore decided to modify the format again and to produce a podcast series instead of a radio programme. This enabled us to more easily define and reach a target audience. It also removed the constraints of the norms and expectations of radio programmes in Uganda and other problems with radio programmes mentioned earlier.

### Choosing the final target audience

We decided to focus on parents of primary school children as our target audience. This would complement the IHC primary school resources [19], with the potential to reinforce learning of both children and their parents, and the potential to reach parents through their children’s schools [27]. The checklist could help clarify that the focus of the podcast was on the IHC key concepts, provide a quick reference and help them to remember the concepts.

Having parents as our target audience enabled us to tailor the podcast to a greater extent, focusing on claims, concepts and stories that would be relevant to this audience. We decided to limit the number of episodes for practical reasons (limited time and resources to produce and evaluate them), but also because we thought that parents might not want to listen to a large number of episodes and we did not want to overwhelm them with too much information. We therefore prioritised nine key concepts using the following procedure: each member of our research team independently made a list of the key concepts they thought we should prioritise for the podcast; we summarised our judgements and discussed disagreements until we arrived at a consensus (Table 8).

**Table 8:** Nine key concepts prioritised for the Informed Health Choices podcast

Recognising an unreliable basis for treatment claims • Treatments may be harmful • Personal experiences or anecdotes (stories) are an unreliable basis for assessing the effects of most treatments • An “outcome” may be associated with a treatment but not caused by the treatment • Widely used treatments or treatments that have been used for a long time are not necessarily beneficial or safe • Opinions of experts or authorities do not alone provide a reliable basis for deciding on the benefits and harms of treatments Understanding whether comparisons are fair and reliable • Identifying effects of treatments depends on making comparisons • Apart from the treatments being compared, the comparison groups need to be similar (i.e. “like needs to be compared with like”) • The results of single comparisons of treatments can be misleading Making informed choices about treatments • Decisions about treatments should not be based on considering only their benefits	

#### Version 2: the IHC podcast

We created a series of eight main episodes, each including a skit in which a claim is made by a character and its trustworthiness is assessed and discussed by other characters. The setting and characters were chosen according to the claim. For example, one episode about the effects of birth control pills was situated at a village health meeting and involved a woman asking a community health worker about the trustworthiness of the claim that birth control pills cause women to gain weight. The skit included an explanation by another character (the community health worker) of why the claim was not trustworthy, by applying an IHC key concept. We used an analogy to help explain the concept, and there was a conclusion with a take-home message. Each main episode was 5–7 min long.

In addition, we developed an introductory episode, 1-min recap episode for every two main episodes, and a conclusion episode, making a total of 13 audio files for each of the two commonly spoken languages (English and Luganda). The recap at the end served as a conclusion, repeating the key messages from all the main episodes. During the user testing, participants listened to the episodes one at a time in their preferred language, and they provided feedback in the same language. Table 6 summarises the contents of each episode, the rationale for the choice of claims and the key concept applied in assessing the trustworthiness of the claim. The complete Version 2 of the IHC podcast can be found here (https://www.youtube.com/playlist?list=PLeMvL6ApG1N35f6DQ3qP9fWE7pMEDZTbH).

Findings from user testing are summarised in Additional file 3. Based on these findings, we clarified that the aim of the podcast was to enable people to make choices, not to tell them what to do. We used the metaphor “give a man a fish and you will feed him for a day; teach a man how to fish and you will feed him for a lifetime” in the introduction episode. In addition, we added a short sentence to the introduction for each episode about the difference between telling them whether a claim is right or wrong and teaching them how to assess any claim. We also edited the introduction to each episode to make them shorter, added voice variations and intonations in those that sounded boring and introduced the claim used in each episode in the introduction to the episode.

Main changes to version 2 of the intervention (the IHC Podcast)

**Table:** 

• Ensuring that men and women were fairly represented in the characters for each episode and that the story and content of each episode would appeal to both men and women	
• Adding more interactive dialogue and distributing talking time more evenly across the characters	
• Correcting all intonations where voices were experienced as flat and ensuring that actors spoke slowly enough for listeners to comprehend	
• Having the characters who learn something in each episode express wanting to share it with others	
• Having a theme song (in both Luganda and English)	
• Replacing the claims used in some of the episodes	
• Clarifying or adding relevant information about the specific claims that were used, such as adding other examples of artemisinin combination treatment (ACT) to episode 1 and adding a message about what you should do when you get a burn to episode 3	
• Improving the explanation of how and why health researchers sometimes compare using a treatment to “no treatment” or to “doing nothing”	
• Improving the explanation of the concept that association is not the same as causation in episode 4	
• Making specific changes to some of the episodes, such as adding restaurant background sounds to episode 3 and changing the setting of episode 4	
• Adding more information to the conclusion episode, including more details from each episode	
• Removing terms that appear for the first time in the conclusion episode	

#### Version 3: the final IHC podcast

In addition to the changes above, we removed the credits to partner institutions in countries other than Uganda from the introduction of each episode. We did this to avoid giving the impression that the messages were coming from a “foreign” place. We emphasised more clearly the difference between this programme and other programmes in every episode by including the statement “In many health programmes people tell you what health choices to make: but in this programme, we explain why some of the things people say about treatments are trustworthy and others are not. If you understand this you can choose for yourself what treatments are right for you”.

To further focus attention on the lessons about assessing claims in each episode, we decided to add a key concept to the introduction of each episode: most treatments of any kind have benefits and harms. Based on our observations of very strong pre-existing beliefs about some claims, we felt that including this key concept in the introduction of each episode would help the audience pay attention to the explanations. We changed the introduction of each episode to include the statement: “most treatments of any type have good effects and bad effects”.

The final podcast can be found here (https://www.informedhealthchoices.org/podcast-for-parents/). It is a series of 13 audio messages teaching nine key concepts that people need to understand and apply in order to assess the trustworthiness of claims about treatments and make informed health choices. This includes an introductory episode, 8 main episodes, 3 recaps to the episodes and a conclusion episode, each produced in two languages. Each main episode lasts 5–7 min, and each recap lasts 1–3 min. The theme song has a mixture of Luganda and English in “Afrobeat”, a popular music genre in Uganda. We incorporated parts of the song at the beginning, in the background and at the end of each episode.

With the help of research assistants and members of our target audience, we explored the feasibility of using small portable media players to deliver the podcast to parents. At the time of development, content such as ours would be challenging to access through internet-based technologies and smartphone access was still very low.

We found that preloading the audio messages onto small inexpensive portable media players and giving these to the parents was a feasible way to deliver the IHC podcast. This also enabled us to circumvent additional problems associated with radio described above. The parents were pleased that they had the opportunity and sufficient time to replay the episodes any time they wished and that the messages on the devices could be shared with others.

## Discussion

We discuss our findings in relation to each of the facets of the honeycomb framework of user-experience [20], focusing on lessons that might be relevant to other researchers working in this and related fields.

### Usefulness

We found increasing appreciation of the usefulness of this work as participants began to understand how much of the “health advice” to which they were exposed was in fact unsubstantiated claims about what we should do to maintain or improve our health. We were informed repeatedly by some editors that if someone can pay for the media space it does not matter what they have to say as long as it is not “politically offensive” or “destructive” to their media enterprise. “It is not our duty to check the trustworthiness of the messages in advertisements”. Most of the people with whom we interacted mistakenly assumed that the government approved all health-related content in the media. People and companies continue to make unsubstantiated claims about the effects of treatments in the mainstream media. Overall, the IHC podcast was seen as a useful tool that could help empower people to question more and assess statements made about the effects of treatments, both in the mass media and elsewhere.

### Usability and understandability

In the early stages of development, we encountered many usability challenges. For example, the print version of the structured press releases lacked important content and the writing style was not acceptable to media houses. The audio version had confusing content, difficult medical terminology, insufficient explanations and a host of production glitches.

People often did not understand the main purpose of the project, and this in turn led to misunderstandings about the content, such as thinking we were going to provide them with health advice. There may be several reasons for this. One is that a lot of people do not routinely question the trustworthiness of treatment claims. When they do, they frequently consider who is making the claim, rather than the basis of the claim. Although fact-checking is common, critically appraising claims about treatment effects is uncommon and is seldom done in a systematic way in the mass media [1, 5, 7–11]. People also question the ability of journalists and nonacademic members of the public to assess the trustworthiness of treatment claims and assume that this is something that requires professional training. We worked with our audiences to adjust each episode, and the approach to the podcast as a whole until they were satisfied that the content was understandable and the podcast was usable.

### Credibility

A few participants in the user testing questioned who was funding this work and who our partners were. Otherwise, we did not find creditability to be a major problem. This may be, in part, because the project was based at Makerere University, which is well known in Uganda. Some journalists, however, did question the source of funding for the project. Concern about funders pushing specific agendas is common in Uganda. We were open about funding sources and the roles of the funders in the project, which helped reassure the journalists. However, we removed credits to non-Ugandan partners in the audio recording so that it would not interfere with the credibility. This information was available for anyone who visited the web site for more details.

### Desirability

Early users (journalists and editors) did not experience most of our initial ideas as desirable (i.e. the structured press releases, the “Be Fair and Compare” news service for journalists and the first version of the Health Choices programme), largely because they perceived these ideas as very unfamiliar and foreign. We abandoned several ideas for this reason.

We changed our target audience, focusing on reaching the public directly, rather than through journalists and editors, and narrowing our target audience to parents of primary school children. We also changed to a story-telling approach to introduce the claims and explain their trustworthiness in version 2 of the IHC podcast, because people tend to make sense of their lives through stories, they hear in the contexts with which they are familiar. A systematic review of the effect of changing health-promoting behaviours through narrative interventions supports the use of a narrative approach [46]. Characters in the narrative can model new behaviours and enhance self-efficacy [47]. Some participants demonstrated the desirability of the podcast by asking if they could have all the episodes so they could listen to them at once or listen more in their own time. A health communication NGO and producers at the Uganda Broadcasting Corporation expressed interest in airing the podcast on radio as part of their health communication programming.

### Identification

Recording in Luganda was likely the most important strategy we employed to create a product that did not feel alienating and foreign to our participants, most of whom had Luganda as their first language and were not fluent in English. We also used terminology, examples of claims, stories and music genres that were appropriate for our target audience. In the earlier versions of the media resources, participants felt that we used unfamiliar language, which alienated them and appeared to reduce their motivation to engage with the content. In one episode, we used a claim which we mentioned was from research done in the Democratic Republic of Congo on the effect of handwashing with soap on reducing diarrhoea. Whereas handwashing is a hygienic practice applicable to Uganda and the whole world, some users experienced the use of an example of a study from Congo as foreign. We also used a claim about Zmapp, which was used to treat American aid workers during the Ebola crisis in western Africa. Again, in addition to Zmapp being a difficult word, the story of Ebola in West Africa was not something with which they could easily identify. We initially used a jazz jingle that people felt was “music for the elite”. We changed that to “Afrobeat”, a music genre most Ugandans listen to.

### Reflexivity

Our own interest was to develop and test our intervention in collaboration with the members of our target audience. We do not know to what extent this might have presented biases in our assessments; however, it is possible that because of project deadlines we might not have explored all the end-users’ feedback to the extent that it might have required. Although we intentionally looked for both negative and positive feedback, our desire to develop useful resources might have led us to interpret some of the feedback more positively or more negatively. Again, we do not know the extent to which this might have been a problem, if at all. Our own positions as researchers from reputable organisations that are well known by the community might have introduced biases in our end-users’ responses, perhaps this might have led them to provide more positive feedback about the resources.

## Conclusions

We have developed an educational podcast to help parents assess the trustworthiness of claims about the effects of treatments. During the design process, we encountered many problems with the early prototypes. We were able to address those problems by working directly with end-users through an iterative, human-centred design approach, which engages end-users in the design process and can help to ensure that an intervention is relevant and acceptable [48]. As a result, we were able to design an educational podcast that listeners experienced as useful, understandable, credible, desirable and appropriate. This, in turn, helped to ensure that our intervention was effective [44]. To our knowledge, the effectiveness of other podcasts for non-formal education or health education have not been evaluated in randomised trials [49–54], and we are unaware of other work describing the use of a similar process to design a mass media intervention to enable people to think more critically about treatment claims.

Because we tailored the podcast to our target audience, it is less likely it will feel familiar to people in other countries. Therefore, the IHC podcast that we developed is unlikely to be transferable to many other contexts. However, others can use the same approach to create a podcast tailored to their target audience, using a guide we have prepared for this purpose [55].

## Supplementary information

**Additional file 1.** Idea generation and selection

**Additional file 2.** Structured press releases and a news service

**Additional file 3.** Findings from user testing the IHC podcast

## Data Availability

All data generated or analysed during this study are included in this published article and its supplementary information files. Any additional raw data will be available from the corresponding author on reasonable request.

## Abbreviation

IHC: Informed Health Choices
