# Supplementary material for: Development of mass media resources to improve the ability of parents of primary school children in Uganda to assess the trustworthiness of claims about the effects of treatments: a human-centred design approach
Source: Pilot Feasibility Stud. 2019 Dec 29;5:155. doi: 10.1186/s40814-019-0540-4 (PMC6935490; doi:10.1186/s40814-019-0540-4)
Supplement: Supplementary file 1 — Additional file 1. Idea generation and selection [file 40814_2019_540_MOESM1_ESM.docx]

**Additional file 1. Idea generation and selection**

**Idea generation**

At the prototyping workshop with the journalist network, the journalists worked in small groups. We led each group through the following process:

1. creating multiple personas using a structured form, then voting on one for each group on which to focus;
2. generating multiple ideas for media channels or venues, then voting on one for each group on which to focus;
3. brainstorming about potential barriers and facilitators for the chosen persona in relation to the chosen media context;
4. generating multiple ideas about specific solutions, then choosing one for each group; and
5. creating a rapid prototype of the chosen idea in a format that demonstrated the idea to the rest of the groups, such as a skit.

Open idea generation by nature has the potential to yield tangential and irrelevant issues. In order to focus our idea generation and increase idea relevance and precision, we used personas, a type of vignette (brief evocative description) and a fictitious character used to present information and provoke thoughts based on the information describing the persona. The personas were descriptions of fictitious people with pre-specified socio-economic and health standing, who for this exercise represented a member of our potential target audience. Personas are also commonly used in human-computer interaction research to help designers understand, focus and clarify users’ goals and behavior patterns.^[[1]](#footnote-1)^

We asked journalists to create personas representing *redacted country’s name* mass media “consumers” of different educational levels based on their own knowledge of their target audiences. In addition to increasing the participants’ focus we used these personas to learn, from the journalists’ perspectives, about our target audiences’ motivations, beliefs, media habits, health information needs and opportunities, prior knowledge and competing sources of information. An example of a persona is shown below.

In addition to ideas for learning-resources, we collected ideas from people in our target audience about relevant treatment claims that we could use as examples when developing resources. We asked members of the journalists’ network and the user-test participants to list claims about the effects of treatments that they had heard of in the recent past.

During the early exploratory phase, we created a Google spreadsheet where we entered all collected ideas. In analysing this data, we understood that not all the information gathered through idea generation would result in actual prototypes. Some would be used for learning about our target audiences’ context, preferences for media content and communication channels, how they use health information, their beliefs, motivations and hindrances. In some cases, multiple suggestions had to be combined to create or support an idea for exploration. Therefore, we took care to identify information that was in and of itself an idea, that which was in support of an idea, that about channels through which a product from an idea could be delivered to the audience, methods for implementing an idea, as well opportunities, barriers and facilitators for an idea, grouping similar ones together and removing duplicates.

**Example of a persona**

**Selection of ideas for prototyping**

We discussed and documented the pros and cons of each idea, tagging them according to their perceived value: which ideas should be retained for further exploration, which should be dropped, and which we were unsure about. This evaluation was informed by a list of desired attributes prepared before collating ideas:

- Credible: Uses trustworthy sources, based on or uses research evidence
- Understandable: Simple to understand, clear straight forward message
- Desirable: Interesting, short, of good quality, memorable
- Contextually appropriate: Contextually, religiously and culturally sensitive
- Feasible: Practical to develop and implement
- Inexpensive: Potential to be developed and implemented with limited resources
- Replicable: Should be easily reproducible
- Transferable: Able to be used in a variety of ways, languages or contexts
- Accessible: Should be easily accessible through conventional means
- Wide coverage: Should have the capacity to reach a large segment of the population

Through multiple iterative cycles of brainstorming, feedback from members of the research team, and discussions with stakeholders, we chose which ideas to prototype, based on how well they fit those attributes. More importantly, they had to be practical and affordable to develop, given our limited budget.

We took notes at every interview and focus group discussion. We also obtained feedback from our journalists’ network multiple times during development. Additionally, all members of the research team provided feedback on the ideas that were generated and the prototypes that we developed throughout the project.

**Example of a persona**

1. Chang Y, Lim Y, Stolterman E. Personas: from theory to practices. In: Proc. 5th Nord. Conf. Human-computer Interact. 2008, 439–442. http://doi.acm.org/10.1145/1463160.1463214. Accessed 3 Aug 2018. [↑](#footnote-ref-1)
