# Supplementary material for: Development of mass media resources to improve the ability of parents of primary school children in Uganda to assess the trustworthiness of claims about the effects of treatments: a human-centred design approach
Source: Pilot Feasibility Stud. 2019 Dec 29;5:155. doi: 10.1186/s40814-019-0540-4 (PMC6935490; doi:10.1186/s40814-019-0540-4)
Supplement: Supplementary file 2 — Additional file 2. Structured press releases and a news service [file 40814_2019_540_MOESM2_ESM.docx]

**Additional file 2. Structured press releases and a news service**

### Structured press releases about treatment claims

The first idea we prototyped was ‘structured press releases’. Our research team developed this idea, informed by our experiences implementing a *Rapid Response Service* for answering policymakers’ urgent needs for reliable evidence.^[[1]](#footnote-1)^ The idea evolved partly in response to the perceived difficulties at the time, associated with developing resources for live radio. It involved preparing contextually appropriate structured press releases^[[2]](#footnote-2)^ in multiple languages, in response to a claim in the media about the effects of a treatment, new research results, or the public’s need for information. The press release would explain in sufficient detail why the claim was trustworthy or not, and provide information about how we applied (and how others could apply) one or more of the IHC Key Concepts to assess the trustworthiness of the claim.

The press releases would be accompanied by packages of materials (also in multiple languages) to provide more detailed explanations or to define any unfamiliar terms. We would provide links to other resources with contextualized scientific evidence, and links to resources that media practitioners and the public could use to learn more about assessing the trustworthiness of treatment claims. We hoped that this would stimulate readers to think critically about related claims about the effects of treatments and help them make more informed health choices. The structured press releases were to be shared for regular publication in the print media.

All versions would be produced in at least two of the most widely spoken languages in *redacted country’s name* (*redacted country’s most commonly spoken language* and English). The press release would be formatted as questions and answers regarding aspects of a reported story that are important for people to understand for them to be able to assess claims about the effects of treatments. The structure included:

- A title, based on the original publication title of the claim in the media
- Sources of the claim about treatments (who said what, where, when? A link to the source)
- Brief heading introducing the claim (“the hook”)
- Outline of the most important issues surrounding the claim
- Why it is important to discuss the claim in our context
- Detailed explanation of the findings from literature relevant to the claim
- Highlights of any issues relating to the local applicability of the findings both from the source of the claim and the evidence used to resolve the claim.
- Guidance on the interpretation of the evidence, in the local context
- Information about how the evidence was gathered and graded
- Links to relevant examples explaining the Key Concept used to explain the trustworthiness of the claim or relating to the terms used in the literature

Box 1 is a prototype of a press release.

| **Box 1. Prototype of a structured press release**  *The following press release was written in response to the following article published 18 February 2014 in the Observer:* [*Energy drinks could be bad for the heart*](http://www.observer.ug/index.php?option=com_content&view=article&id=30209:-energy-drinks-could-be-bad-for-the-heart&catid=58:health-living&Itemid=89)  **Press release**  No reason to worry about energy drinks being bad for your heart   - **What’s the hook?**   Should you be worried about using energy drinks? A recent study reported in the Observer suggests maybe you should be.   - **What’s the claim?**   Energy drinks could be bad for the heart   - **What’s the bottom line?**   ***The results aren’t relevant.*** Energy drinks may or may not have other good or bad effects, but there is not reliable evidence that they are bad for the heart.   - **What’s the basis for the claim?**   A study that showed that energy drinks high in caffeine and taurine (an amino acid said to enhance athletic performance) increased heart rates by six per cent in the 18 people that were part of the study.   - **Has all the reliable evidence been considered?**   No. There may be other, better studies that have addressed this question.   - **Are the results relevant?**   No. The outcome that was measured (heart rates) is a ***surrogate outcome*** (see below). It is not of direct practical importance. If energy drinks cause a small increase in heart rate, that doesn’t mean they are bad for your heart.   - **Has the treatment been tested fairly?**   Can’t tell.   - **What are the results?**   Uninteresting   - **What’s the likelihood of being misled by the play of chance?**   In addition to not measuring outcomes that are important to people, the study only included 18 people. That might be enough to measure a change in heart rate, but it is not enough to measure important effects, including presumed benefits – like better athletic performance, increased concentration and maintaining alertness – and presumed adverse effects – like heart disease, addiction, anxiety and insomnia.  **Resource**  Surrogate outcomes  Surrogate outcomes are outcome measures that are not of direct practical importance but are believed to reflect outcomes that are important.  For example, blood pressure is not directly important to patients but it is often used as an outcome in studies because it is a risk factor for stroke and heart attacks. Showing that a medicine lowers blood pressure is not the same as showing that it prevents strokes or heart attacks.  ***The effects of treatments on surrogate outcomes usually do not provide a reliable indication of effects on outcomes that are important.***  For more information see . . . [link to visualization/text or audio clips of examples of how relying on surrogate outcomes has caused harm (e.g. antiarrhythmic drugs for heart attacks) + maybe when a surrogate outcome is reliable (e.g. smoking)] |
| --- |

##### We conducted a focus group discussion with journalists and editors to explore how they experienced the prototypes. Although they experienced the press releases as short, easy to read and straight forward, they had several concerns. The largest concern was that journalists would likely experience the structured press releases as being critical of their reporting instead of helping to make it better. The writing style was very unfamiliar to the journalists, and the press releases were too brief and lacking information.

We dropped this idea because of our perception that it would be difficult to get stories based on the press releases published.

### The “Be Fair and Compare” News Service

To address the barriers to structured press releases, we prototyped a service that would prepare full stories in a format that would appeal to audiences. The “Be Fair and Compare” News Service was proposed as a news wire service based at *redacted institution’s name*, the largest and oldest medical school and health research institution in the country. The idea was to provide a high volume of brief, ready-to-use articles and audio files that the mainstream media could publish directly. The stories would be based on claims about effects of treatments deemed of interest to the public. These would be identified and collated by scanning the media, monitoring new research, and interviewing members of the public.

We developed both an audio and a print prototype. The audio prototype was developed as a short, pre-recorded audio message that included a claim about the effectiveness of Zmapp for treating Ebola virus disease. It started with opinions of ordinary people about the trustworthiness of the claim before they listened to any explanations. This was followed by an explanation of why the claim was not trustworthy using an IHC Key Concept, and an explanation of the concept. It concluded with opinions of the same people after they had listed to the explanation, and a conclusion. At the time of production of our audio prototype Zmapp was still an investigational drug early in development. Claims that it was effective were based on anecdotal evidence. The audio prototype can be found [here.](https://youtu.be/amWxsZ0Y130)

The print prototype was designed to have a catchy title, a “hook” (a good introduction) to get people interested in reading the story, the claim and important issues to consider when assessing the reliability of the claim. The claim used in the prototype was based on an article that appeared in local newspapers stating that a drug called “Canova” was effective in ameliorating the effects of HIV.^[[3]](#footnote-3)^ Box 2 is the print prototype.

| **Box 2. Prototype of a print story**  **BE FAIR and COMPARE *NEWS***  ***A MakHealth Initiative***  **Is Canova a wonder drug for HIV/AIDS?**  *By redacted author’s name Health Researcher, redacted country’s name*  Recently, in New Vision, there was a story about a Ugandan woman who made an amazing recovery from HIV/AIDS. The woman’s cousin brought her Canova and within a month of starting to use the drug, the woman was back at work, according to the report. But what do we really know about the effect of Canova on HIV/AIDS?  Doctors interviewed in the report say Canova is easy to use, prolongs the life of HIV/AIDS patients and improves their quality of life. Apparently, Canova has no side effects either. If this is all true Canova is, indeed, a wonder drug, but the truth is rarely that simple.  There are many problems with the claim that Canova is a wonder drug, but let us focus on one: why treatments rarely work well for everyone all the time.  To understand why treatments rarely work well for everyone all the time, imagine a mechanic told us he had built an engine that works well for every car.  Is it likely that the mechanic has built an engine that works well for every car?  No, car parts that work well for every car are rare!  Cars are complicated machines and no two models are exactly alike in form or function. Some car parts are almost the same for most cars and work well for most, for example windshield wipers. These parts are rare. It is common for parts to be different from model to model, for example engines, and work well for some cars some times, but not all cars all the time. This is why it is unlikely the mechanic has built an engine that works well for every car.  The same goes for medicine. Just like models of cars, no two bodies are exactly alike and bodies are even more complicated than cars. This is why treatments that work well for everyone all the time are so rare, which means it is unlikely that Canova is a wonder drug.  To know if Canova is a wonder drug, the effect of using the drug needs to be judged in a fair comparison. We need to be fair and compare!  For more information about the [Be Fair and Compare project](http://www.bf&c.org) and how you can be fair and compare, send an SMS with the text “BFC” to [#]. You can also find more information on our Facebook page at www.facebook.com/bfcug. |
| --- |

We organized a focus group discussion with four editors of major news organizations in *redacted country’s name* one journalist, and one health communication specialist to explore how they experienced the prototype and the proposed news service. They listened to the audio prototype and read the print prototype before we started the discussion. In addition, we carried out individual face-to-face interviews with four random members of the non-academic public to explore how they experienced the prototypes. They also listened to the one and read the other prototype before being interviewed.

All the participants in the focus group were positive and expressed interest in the general idea due to, in their own words, the need for “evidence-based healthcare”, “improving science writing”, “simplification of health research” and “dispelling misinformation about treatments.” However, the idea of enabling their audiences to assess the trustworthiness of media content was a totally new concept to the editors. They wanted to provide their audiences with clear messages and definitive answers regarding the reliability of the treatment claim. As one editor put it; *“experts should be available to provide precise answers.”* They also were concerned about stories not vetted by them. They believed that their role as gatekeepers on behalf of their audiences was important, and they were sceptical about the idea of improving their audiences’ ability to vet information (or claims about treatments) themselves.

Most of the positive feedback was about the style of presenting information. Participants liked the beginning of the audio prototype which had voices of people on the street being asked about their opinions regarding the claim. They liked our approach of explaining complex health information using analogous illustrations, and they thought that this was clearer in the audio prototype than the print prototype.

However, some editors had the impression that we were the ones making the claim about *Canova* while others thought that we left the audience with the impression that *Canova* is effective as stated in the claim. Some thought the aim of the project was to provide research results. Several wanted information that we thought was irrelevant to addressing claims, such as how drugs are approved or how they work.

Participants thought that the stories did not have adequate details about the source of the claim, the claim itself, the assessment of the claim and the explanatory information. *“Unfamiliar terms were not explained e.g. experimental drugs, Zmapp”* (Public participant 1); *“The message seems incomplete, hanging – should inform people about what they should do. It is not enough to just tell people to compare and be fair or send SMS. There should be a conclusion about how to interpret the information in the claim”* (Public participant 2). The editors suggested that feature articles would work better than short articles. One editor suggested that we consider using pull-out supplements inserted in newspapers.

The focus group thought that there were too many documents in the “Be Fair and Compare News Service operating procedures and Resources” folder (templates and guidelines), which we showed them. They thought there was too much information in those documents to write hard news stories without missing information or compromising the newsworthiness and entertainment value of the story. They felt it was not obvious which document to use and when. It was also confusing that some documents applied to only some stories e.g. the SUPPORT summary checklist,^[[4]](#footnote-4)^ [33] which we had developed for use if the claim based on findings of a systematic review of the effects of a treatment.

The editors believed that the public might not be able to access sources of information through the links we included. They mentioned that *“social media platforms are for the elite.”* However, some thought SMS messaging was growing rapidly and could be a valuable communication modality as part of the project. They said they would not publish any reports or links in news stories prepared by our project as such material would be considered commercial, or any public service announcement, as it was their expectation that those should be paid for. Furthermore, they said that all our stories would be subjected to editorial review, and that they would be unlikely to publish them without first revising them, if they published them at all.

The four members of the non-academic public (representatives of our target audience) also generally found the prototypes confusing and they said they would find it hard to use Facebook and SMS, because there are costs involved. With Facebook one would need a smart phone, which is very costly and SMS text messages cost money. Furthermore, some said the print prototype would be difficult to access for people who can’t read.

1. Mijumbi-Deve R, Rosenbaum SE, Oxman AD, Lavis JN, Sewankambo NK. Policymaker experiences with rapid response briefs to address health-system and technology questions in Uganda. Health Syst Policy Res 2017, 15:37. [↑](#footnote-ref-1)
2. Woloshin S, Schwartz LM. Press releases: translating research into news. JAMA 2002, 287:2856–2858. [↑](#footnote-ref-2)
3. Bainemigisha H. New wonder drug for HIV/AIDS. In New Vision, 2 Aug 2005. <http://www.newvision.co.ug/new_vision/news/1119976/wonder-drug-hiv-aids>. Accessed 3 Aug 2018. [↑](#footnote-ref-3)
4. Rosenbaum SE, Glenton C, Wiysonge CS, Abalos E, Mignini L, Young T, et al. Evidence summaries tailored for health policymakers in low and middle-income countries. WHO Bull 2011, 89:54–61. [↑](#footnote-ref-4)
