# Supplementary material for: Development of mass media resources to improve the ability of parents of primary school children in Uganda to assess the trustworthiness of claims about the effects of treatments: a human-centred design approach
Source: Pilot Feasibility Stud. 2019 Dec 29;5:155. doi: 10.1186/s40814-019-0540-4 (PMC6935490; doi:10.1186/s40814-019-0540-4)
Supplement: Supplementary file 3 — Additional file 3. Findings from user testing the IHC podcast [file 40814_2019_540_MOESM3_ESM.docx]

**Additional file 3. Findings from user testing the IHC podcast**

##### More participants preferred listening to the episodes in Luganda than English. Even participants that listened in English suggested that we should have a version of the same messages in other languages. *“The messages should be translated to Luganda and other vernacular languages that most people in Uganda understand” (Participant 11).*

Overall, participants’ comments were positive for most of the episodes and for the series as a whole. Most participants who had listened to this and previous versions liked the new structure and flow of each episode, the introduction, the claims, the explanations, the discussions, the characters and the jingles.

"*I enjoyed the fact that Mr. Sonko wants to share what he learned from the explanations. But let him say he wants to share with the community in instead of sharing with only his wife. This message is good and should not just be shared with only one person"* (Participant 03, episode 6).

Most participants thought that the claims we used in the episodes were appropriate.

*“The radio episode has an interesting message because it’s about malaria which affects a lot of people”* (Participant 02, episode 1).

Many seemed to understand the main message and for most, claims were no longer being confused for the key messages.

*"The claim used is good, very simple and very common. A lot of people have heard many claims about ulcer treatments. This was good"; “The audio clip is about how much one should trust experts. Is your trust based on research? Even though you enquire from experts about treatments you have to first find out if what they say is from scientific research"* (Participant 01, episode 6).

*“This is Ok. The doctor tried to explain the importance of numbers in carrying out research.”* (Participant 03, episode 8)

*“I have learnt that medicine is not 100% perfect because it has its other effects”.* (Participant 17, episode 1)

*“The part I didn’t understand was why health workers used Zmapp when it was not tested.”* (Participant 15, episode 2)

However, some participants still thought that the purpose of the project was to provide specific messages about what people should do to improve their health. Some wanted more general information about the health conditions we presented in the episodes while others wanted more information about the treatments we mentioned and how they work.

*“The episode should talk about a disease, how it is transmitted and how it can be cured” (Participant 19);* *“The episode should be made clearer and, also tell us the cause of Ebola” (Participant 09)*; *“The conclusion should at least sum up with the signs of Ebola inclusive” (Participant 18).*

*“The project should sensitize people more about other methods that can prevent AIDS” (Participant 21);* *“People should be enlightened about any other measures to HIV-AIDS prevention apart from circumcision alone” (Participant 18)*.

Some participants observed that the message about what health researchers should do to be more certain about the effects of treatments came across as simply a preference of some health researchers [instead of the best way to determine the effects of treatments]. This was a statement about the need for fair comparisons – randomized controlled trials of treatments as the best way of evaluating the effects of treatments.

Some participants were still missing the main message even though it was being repeated for every episode. Others were still confused by the details of the explanations.

*“The other part that confused me was the method of comparing a treatment with no treatment. I did not understand it clearly”* (Participant 13, all episodes).

*“I did not understand why large numbers are used to determine whether a treatment is effective*;” (Participant 14, last 3 episodes)

*“I did not really understand the terms association and causation;”* (Participant 25, episode 4)

*“I Did not understand the claim about babies sleeping on their stomachs”* (Participant 06, episode 5)

Some people felt that they would need a lot more time to understand the main message of some episodes. As participant 06 put it: *“Understanding the message in this episode would need a very long time because it’s confusing;”* (about episode 5). One person wondered how one can tell if a comparison of treatments was *“big enough”* (Participant 12).

Some participants felt that the two examples we used in each episode to explain the Key Concepts within each episode should have worked better if they were more closely related.

*“Both examples confused me. The episode started with a claim on sleeping positions for babies and ended with use of soil for treating HIV/AIDS. There was no take home message because I saw that the two claims were not corresponding.”* (Participant 19, episode 5)

*“The examples are not appropriate. The example of “boda bodas (motor cycle taxis)” is not related to the one for children and diarrhea.”* (Participant 21, episode 8)

Some participants mentioned that some of the examples we had used were difficult to understand and probably inappropriate for the context. The example about Zmapp, the investigational drug for treating Ebola that we used in episode 2 was noted to be unfamiliar to most participants even though it was talked about quite often in international media.

*“The claim about Ebola was not well known to me and to the community where I stay.”* (Participant 13, episode 2) Another commented that the example used in episode 8 was out of context: *“The example given was outside the bounds of the project because it was in Congo.”* (Participant 04).

In addition, many participants had very strong opinions about some of the claims and examples we used in version 2 to the extent that they became a distraction. Of particular concern was the claim used in episode 7: medical male circumcision for preventing HIV.

*“The comparison was wrong. Circumcision doesn't prevent AIDS but maybe other diseases. Circumcision does not stop one from getting AIDS.”* (Participant 24)

*"You should use another example. The circumcision and HIV prevention was confusing. There are many people who are circumcised who have HIV. This message will be mixed up.” (Participant 04).*

Some terms used in the episode were unfamiliar and elicited a range of differing reactions. While several user-test participants didn’t understand the acronym “ACTs” and found it confusing, some participants found the accompanying examples such as Coartem® which people already knew, helpful.

*“What is ACTs? This does not make the episode clear. Define ACTs in simple terms.”* (Participant 04, episode 1)

*“The message ACT, is a put off, it sounds like a message for doctors”* (Participant 03, episode 1)

*“I would not pay much attention to the episode because some of the words used like Zmapp were not familiar” (Participant 13, episode 2)*

*“I can’t remember the claim. It was a bit confusing. Z..something, Zmapp is confusing. Never heard of it!”* (Participant 02)

We had used the term “an experience” to mean “personal stories about one’s experience receiving a treatment” however, some people confused this as meaning “having experience” in doing something.

Participants were divided on the issue of length. Some participants felt that the episodes were still too long while others felt it was of the appropriate length given the needed explanations and examples.

*“Reduce on the length of episodes i.e. the introduction and conclusion are too long. We can do without the introduction”* (Participant 17, episode 4).

*“The episode is lengthy. One can forget about the other information.”* (Participant 19, episode 1)

*“The clip of the episode is lengthy therefore reduce on the announcer's time and use at least one example that can be understood easily.”* (Participant 17, episode 5).

There were several comments about specific details that might improve the overall outlook of the program and users’ experience listening, for example:

*“The two characters were very good, but the third character – the doctor needed to be more serious in the episode and should articulate his words in a better way.”* (Participant 09, episode 7)

Participant 21 observed that he could not differentiate the three voices of the actor in episode 8, and some mentioned that talking time was not balanced among the characters in the same episode:

*“the shopkeeper at some point vanished from the discussion. He should be given more time”* (Participant 02, episode 8).
